# Crystal structure of an eight-coordinate terbium(III) ion chelated by *N*,*N*′-bis­(2-hy­droxy­benz­yl)-*N*,*N*′-bis­(pyridin-2-ylmeth­yl)ethyl­enedi­amine (bbpen^2−^) and nitrate

**DOI:** 10.1107/S2056989014026826

**Published:** 2015-01-01

**Authors:** Thaiane Gregório, André Luis Rüdiger, Giovana G. Nunes, Jaísa F. Soares, David L. Hughes

**Affiliations:** a Departamento de Química, Universidade Federal do Paraná, Centro Politécnico, Jardim das Américas, 81530-900 Curitiba-PR, Brazil; b School of Chemistry, University of East Anglia, Norwich NR4 7TJ, England

**Keywords:** crystal structure, lanthanide, terbium(III), *N,N*′-bis(2-hy­droxy­benz­yl)-*N*,*N*′-bis­(pyridin-2-ylmeth­yl)ethyl­enedi­amine, mononuclear, dodeca­hedral.

## Abstract

The reaction of terbium(III) nitrate penta­hydrate in aceto­nitrile with *N*,*N*′-bis­(2-hy­droxy­benz­yl)-*N*,*N*′-bis­(pyridin-2-ylmeth­yl)ethyl­enedi­amine (H_2_bbpen), previously deprotonated with tri­ethyl­amine, produced the mononuclear compound [*N*,*N*′-bis­(2-oxidobenzyl-κ*O*)-*N*,*N*′-bis­(pyridin-2-ylmethyl-κ*N*)ethylenedi­amine-κ^2^ *N*,*N*′](nitrato-κ^2^ *O*,*O*′)terbium(III), [Tb(C_28_H_28_N_4_O_2_)(NO_3_)]. The mol­ecule lies on a twofold rotation axis and the Tb^III^ ion is eight-coordinate with a slightly distorted dodeca­hedral coordination geometry. In the symmetry-unique part of the mol­ecule, the pyridine and benzene rings are both essentially planar and form a dihedral angle of 61.42 (7)°. In the mol­ecular structure, the N_4_O_4_ coordination environment is defined by the hexa­dentate bbpen ligand and the bidentate nitrate anion. In the crystal, a weak C—H⋯O hydrogen bond links mol­ecules into a two-dimensional network parallel to (001).

## Abstract 2

The reaction of terbium(III) nitrate penta­hydrate in aceto­nitrile with *N*,*N*′-bis­(2-hy­droxy­benz­yl)-*N*,*N*′-bis­(pyridin-2-ylmeth­yl)ethyl­enedi­amine (H_2_bbpen), previously deprotonated with tri­ethyl­amine, produced the mononuclear compound [Tb(Cbbpen)(NO_3_)]. The mol­ecule lies on a twofold rotation axis and the Tb^III^ ion is eight-coordinate with a slightly distorted dodeca­hedral coordination geometry.

## Chemical context   

As far as biological and biomedical applications are concerned, complexes of polydentate ligands with a range of metal ions in different oxidation states have been synthesized to model active sites of metalloproteins and to shed light on the consequences of heavy-metal chelation in living organisms, among many other applications (Colotti *et al.*, 2013 ▸; Nurchi *et al.*, 2013 ▸; Sears, 2013 ▸; Happe & Hemschemeier, 2014 ▸). Pyridyl and phenolate groups have been incorporated into these ligands because of their potential to mimic the coordination environments provided by the amino acids histidine and tyrosine, respectively (Hancock, 2013 ▸; Lenze *et al.*, 2013 ▸). In this context, the heterotrifunctional Lewis base *N*,*N*′-bis­(2-hy­droxy­benz­yl)-*N*,*N*′-bis­(pyridin-2-ylmeth­yl)ethyl­enedi­amine (H_2_bbpen) is suitable for the coordination of a range of *p-*, *d-* and *f-*block ions because of its versatile soft donor atoms in the pyridine rings and hard donors in the amine and phenolate groups (Neves *et al.*, 1992 ▸; Schwingel *et al.*, 1996 ▸). Electrochemical studies of the mononuclear [Mn(bbpen)]PF_6_, for example, revealed that this complex mimics some of the redox features of the photosystem II (PSII) (Neves *et al.*, 1992 ▸). Complexes of bbpen^2–^ with vanadium(III) and oxido-vanadium(IV) have been obtained as models of the vanadium-modified transferrin, the probable vanadium-transporting protein in higher organisms (Neves *et al.*, 1991 ▸, 1993 ▸). Iron complexes of bbpen^2–^ modified with electron-donating and -withdrawing groups (Me, Br, NO_2_), in turn, have been synthesized to provide detailed chemical information on the enzymatic activity of iron-tyrosinate proteins (Lanznaster *et al.*, 2006 ▸). This ligand has also been employed to prepare lanthanide(III), gallium(III) and indium(III) complexes for medicinal applications such as the development of new contrast agents for magnetic resonance imaging, MRI (Wong *et al.*, 1995 ▸, 1996 ▸; Setyawati *et al.*, 2000 ▸).
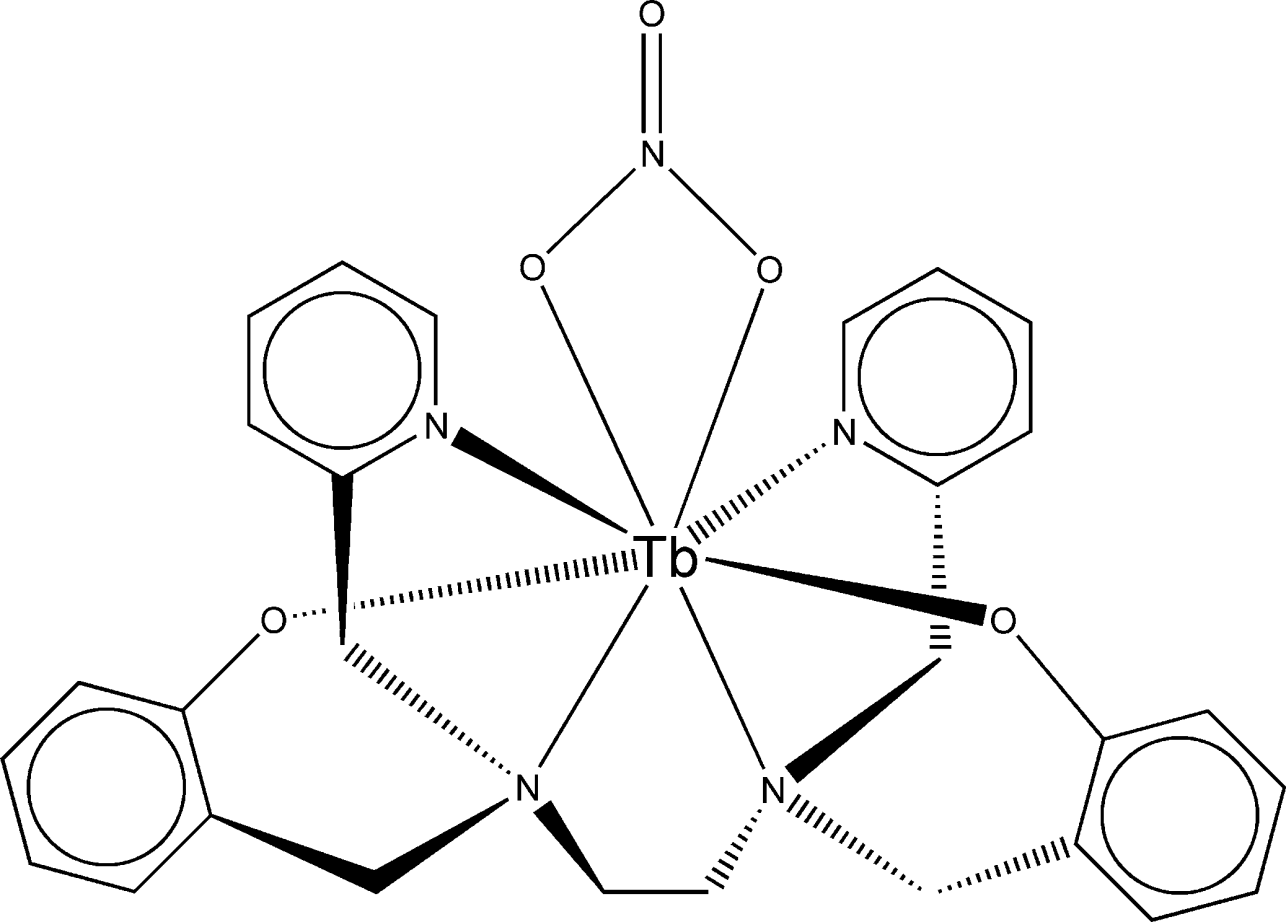


More recently, lanthanide(III) chelate complexes have also attracted attention in the field of mol­ecular magnetism due to their highly significant single-ion magnetic anisotropy (Sessoli & Powell, 2009 ▸; Luzon & Sessoli, 2012 ▸). Accordingly, a number of examples of mononuclear lanthanide complexes that exhibit single-mol­ecule magnet (SMM) behaviour have been reported (Rinehart & Long, 2011 ▸; Chilton *et al.*, 2013 ▸; Ungur *et al.*, 2014 ▸; Zhang *et al.*, 2014 ▸). Our inter­est in the class of lanthanide complexes in which two coordination sites are occupied by relatively labile ligands, as in the title complex, comes from the possibility of using them as starting materials for the preparation of heteronuclear aggregates of *d-* and *f-*block ions that present SMM features. In this case, the replacement of the labile ligands by specific bidentate metallo­ligands can give rise to heteronuclear metal aggregates in which desirable ferromagnetic or ferrimagnetic exchange inter­actions are favoured (Totaro *et al.*, 2013 ▸; Westrup *et al.*, 2014 ▸).

## Structural commentary   

**Figure 1:**
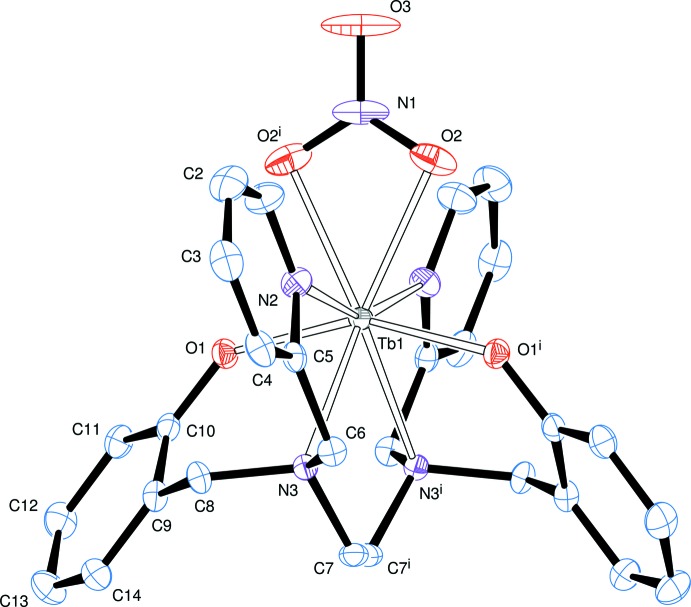
View of a mol­ecule of [Tb(bbpen)(NO_3_)], indicating the atom-numbering scheme. H atoms have been omitted for clarity. Displacement ellipsoids are drawn at the 50% probability level [symmetry code: (i) −*x* + 1, *y*, −*z* + 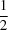].

The mol­ecular structure of the title compound is shown in Fig. 1 ▸. The Tb^III^ ion is eight-coordinate with a dodeca­hedral array of N and O atoms (Table 1 ▸); the four N atoms of the O_2_N_4_-ligand (bbpen) form one plane, the four O atoms the other, with the phenolic O atoms in the B-sites (roughly equatorial) and the nitrate group O atoms in the A-sites (above and below the equatorial plane). The normals to the two planes are essentially perpendicular. A twofold rotation axis passes through O3 and N1 of the nitrate group, the terbium(III) atom and the mid-point of the C7—C7^i^ bond [symmetry code (i) 1 − *x*, *y*, −*z* + 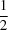]. In the symmetry-unique part of the mol­ecule, the pyridine and benzene rings are both essentially planar and form a dihedral angle of 61.42 (7)°. The eightfold coordination pattern might also be described as a distorted bicapped trigonal prism with O1 and N2 as the capping atoms. However, this ignores the symmetry of the coordination, *e.g.* O1 and O1^i^ would occupy different sites in the coordination polyhedron. Also, some of the rectangular faces of the prism are difficult to identify. In contrast, the dodeca­hedral pattern incorporates the twofold symmetry and the distortion from the ideal geometry is minimal.

## Supra­molecular features   

**Figure 2:**
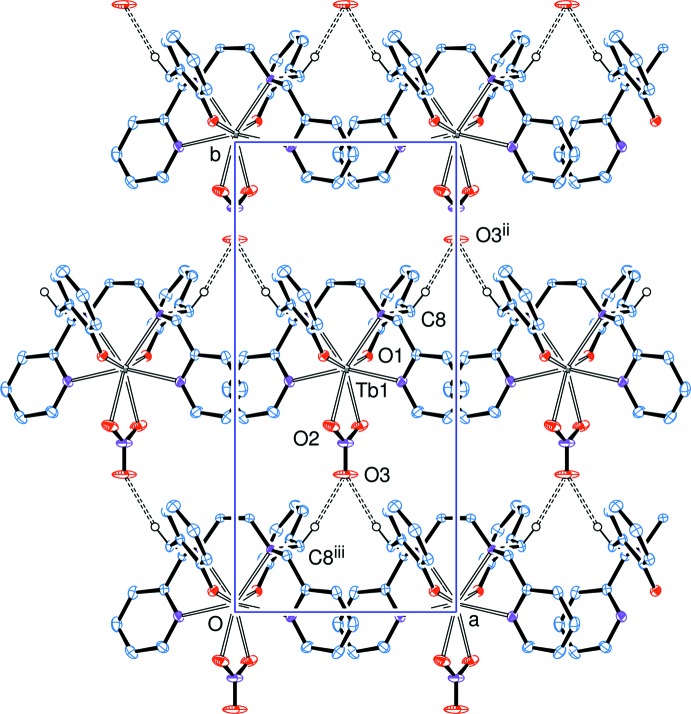
A sheet of mol­ecules, lying in a plane normal to the *c* axis, linked through short ‘weak hydrogen bonds’, as C8—H8*B*⋯O3^ii^ [symmetry codes: (ii) *x* + 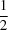, *y* + 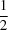, *z*; (iii) *x* − 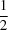, *y* − 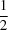, *z*].

In the crystal, a weak C—H⋯O hydrogen bond (Table 2 ▸) links mol­ecules into a two-dimensional network parallel to (001), Fig. 2 ▸.

## Database survey   

Some examples of complexes with bbpen^2–^ and related ligands with *d-*block metal ions appear in the literature (Xu *et al.*, 2000 ▸; dos Anjos *et al.*, 2006 ▸; Lanznaster *et al.*, 2006 ▸; Golchoubian & Gholamnezhad, 2009 ▸; Thomas *et al.*, 2010 ▸) as well as *p-*block metal(III) compounds (Wong *et al.*, 1995 ▸, 1996 ▸) and related yttrium(III) and lanthanide(III) complexes (Setyawati *et al.*, 2000 ▸; Yamada *et al.*, 2010 ▸).

## Synthesis and crystallization   

Tb(NO_3_)_3_·5H_2_O, ethyl­enedi­amine, salicyl­aldehyde, sodium borohydride, 2-picolyl-chloride hydro­chloride and tri­ethyl­amine were purchased from Aldrich and used without purification. *N*,*N′*-bis­(salicyl­idene)ethyl­enedi­amine (H_2_salen) (Diehl *et al.*, 2007 ▸), *N*,*N′*-bis­(2-hy­droxy­benz­yl)ethyl­enedi­amine (H_2_bben) and *N*,*N′*-bis­(2-hy­droxy­benz­yl)-*N*,*N*′-bis(2-pyridyl­meth­yl)ethyl­enedi­amine (H_2_bbpen) (Neves *et al.*, 1992 ▸) were prepared as described in the literature. The preparation of the title complex was carried out under N_2_(g) using standard Schlenk and glove-box techniques. Aceto­nitrile was dried with CaH_2_ and distilled prior to use. A solution containing tri­ethyl­amine (300 µl, 2.15 mmol) in aceto­nitrile (10 ml) was added to a suspension of H_2_bbpen (0.454 g, 1.00 mmol) in aceto­nitrile (25 ml) under stirring, giving a clear light-orange solution. After 15 min, this solution was added to a colourless solution of Tb(NO_3_)_3_·5H_2_O (0.434 g, 0.998 mmol) in aceto­nitrile (25 ml). A pale-yellow solution was obtained, which gave a 65% yield of the solid of the title compound upon cooling at 253 K for 2–3 days. Recrystallization of this solid by vapor diffusion of di­meth­oxy­ethane into the reaction mixture gave pale-pink crystals after two weeks at room temperature. These crystals are air-stable and insoluble in all common organic solvents.

## Refinement   

Crystal data, data collection and structure refinement details are summarized in Table 3 ▸. Hydrogen atoms were included in idealized positions (with C—H distances set at 0.97 and 0.93 Å for the methyl­ene and trigonal–planar groups, respectively) and their *U*_iso_ values were set to ride (1.2×) on the *U*_eq_ values of the parent carbon atoms.

## Supplementary Material

Crystal structure: contains datablock(s) I. DOI: 10.1107/S2056989014026826/lh5741sup1.cif

Structure factors: contains datablock(s) I. DOI: 10.1107/S2056989014026826/lh5741Isup2.hkl

CCDC reference: 1037922

Additional supporting information:  crystallographic information; 3D view; checkCIF report

## Figures and Tables

**Table 1:** Selected bond lengths ()

Tb1O1	2.1947(13)	Tb1N3	2.5558(16)
Tb1O2	2.4764(15)	Tb1N1	2.891(2)
Tb1N2	2.5521(17)		

**Table 2:** Hydrogen-bond geometry (, )

*D*H*A*	*D*H	H*A*	*D* *A*	*D*H*A*
C8H8*B*O3^ii^	0.99	2.37	3.338(3)	166

Symmetry code: (ii) 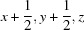.

**Table 3:** Experimental details

Crystal data	
Chemical formula	[Tb(C_28_H_28_N_4_O_2_)(NO_3_)]
*M*_r_	673.47
Crystal system, space group	Orthorhombic, *C*222_1_
Temperature (K)	100
*a*, *b*, *c* ()	8.5947(6), 18.2401(17), 16.9272(13)
*V* (^3^)	2653.6(4)
*Z*	4
Radiation type	Mo *K*
(mm^1^)	2.71
Crystal size (mm)	0.43 0.20 0.20

Data collection	
Diffractometer	Bruker D8 Venture/Photon 100 CMOS
Absorption correction	Multi-scan (*SADABS2014/2*; Bruker, 2014 ▸)
*T*_min_, *T*_max_	0.581, 0.746
No. of measured, independent and observed [*I* > 2(*I*)] reflections	75009, 3320, 3289
*R*_int_	0.020
(sin /)_max_ (^1^)	0.668

Refinement	
*R*[*F* ^2^ > 2(*F* ^2^)], *wR*(*F* ^2^), *S*	0.010, 0.027, 1.15
No. of reflections	3320
No. of parameters	178
H-atom treatment	H-atom parameters constrained
_max_, _min_ (e ^3^)	0.87, 0.30
Absolute structure	Flack *x* determined using 1431 quotients [(*I* ^+^)(*I* )]/[(*I* ^+^)+(*I* )] (Parsons Flack, 2004 ▸)
Absolute structure parameter	0.0107(19)

Computer programs: *APEX2* and *SAINT* (Bruker, 2010 ▸), *SHELXS97* and *SHELXL2013* (Sheldrick, 2008 ▸), *ORTEPII* (Johnson, 1976 ▸), *ORTEP-3 for Windows* and *WinGX* (Farrugia, 2012 ▸).

## supplementary crystallographic information

### Crystal data

**Table:**

[Tb(C_28_H_28_N_4_O_2_)(NO_3_)]	*D*_x_ = 1.686 Mg m^−^^3^
*M**_r_* = 673.47	Mo *K*α radiation, λ = 0.71073 Å
Orthorhombic, *C*222_1_	Cell parameters from 9558 reflections
*a* = 8.5947 (6) Å	θ = 2.9–28.3°
*b* = 18.2401 (17) Å	µ = 2.71 mm^−^^1^
*c* = 16.9272 (13) Å	*T* = 100 K
*V* = 2653.6 (4) Å^3^	Prism, pale pink
*Z* = 4	0.43 × 0.20 × 0.20 mm
*F*(000) = 1344	

### Data collection

**Table:**

Bruker D8 Venture/Photon 100 CMOS diffractometer	3320 independent reflections
Radiation source: fine-focus sealed tube	3289 reflections with *I* > 2σ(*I*)
Graphite monochromator	*R*_int_ = 0.020
Detector resolution: 10.4167 pixels mm^-1^	θ_max_ = 28.4°, θ_min_ = 2.9°
φ and ω scans	*h* = −11→11
Absorption correction: multi-scan (*SADABS2014*/2; Bruker, 2014)	*k* = −24→24
*T*_min_ = 0.581, *T*_max_ = 0.746	*l* = −22→22
75009 measured reflections	

### Refinement

**Table:**

Refinement on *F*^2^	Hydrogen site location: inferred from neighbouring sites
Least-squares matrix: full	H-atom parameters constrained
*R*[*F*^2^ > 2σ(*F*^2^)] = 0.010	*w* = 1/[σ^2^(*F*_o_^2^) + (0.0139*P*)^2^ + 1.0428*P*] where *P* = (*F*_o_^2^ + 2*F*_c_^2^)/3
*wR*(*F*^2^) = 0.027	(Δ/σ)_max_ = 0.004
*S* = 1.15	Δρ_max_ = 0.87 e Å^−^^3^
3320 reflections	Δρ_min_ = −0.30 e Å^−^^3^
178 parameters	Absolute structure: Flack *x* determined using 1431 quotients [(*I*^+^)-(*I*^-^)]/[(*I*^+^)+(*I*^-^)] (Parsons & Flack, 2004)
0 restraints	Absolute structure parameter: −0.0107 (19)
Primary atom site location: structure-invariant direct methods	

### Special details

**Table:**

Geometry. All e.s.d.'s (except the e.s.d. in the dihedral angle between two l.s. planes) are estimated using the full covariance matrix. The cell e.s.d.'s are taken into account individually in the estimation of e.s.d.'s in distances, angles and torsion angles; correlations between e.s.d.'s in cell parameters are only used when they are defined by crystal symmetry. An approximate (isotropic) treatment of cell e.s.d.'s is used for estimating e.s.d.'s involving l.s. planes.

### Fractional atomic coordinates and isotropic or equivalent isotropic displacement parameters (Å^2^)

**Table:**

	*x*	*y*	*z*	*U*_iso_*/*U*_eq_	
Tb1	0.5000	0.51826 (2)	0.2500	0.01172 (4)	
O1	0.59854 (16)	0.54526 (8)	0.13398 (8)	0.0167 (3)	
O2	0.4357 (2)	0.39605 (9)	0.30473 (10)	0.0312 (4)	
O3	0.5000	0.29285 (11)	0.2500	0.0586 (9)	
N1	0.5000	0.35974 (11)	0.2500	0.0293 (6)	
N2	0.7540 (2)	0.49068 (9)	0.32221 (10)	0.0183 (3)	
N3	0.66305 (19)	0.63257 (8)	0.27777 (9)	0.0130 (3)	
C1	0.8272 (3)	0.42575 (12)	0.32538 (14)	0.0249 (4)	
H1	0.7809	0.3851	0.2993	0.030*	
C2	0.9672 (2)	0.41490 (13)	0.36483 (14)	0.0275 (5)	
H2	1.0155	0.3681	0.3656	0.033*	
C3	1.0342 (2)	0.47389 (13)	0.40282 (14)	0.0253 (5)	
H3	1.1304	0.4685	0.4298	0.030*	
C4	0.9590 (2)	0.54124 (13)	0.40111 (12)	0.0203 (4)	
H4	1.0022	0.5823	0.4278	0.024*	
C5	0.8200 (2)	0.54783 (11)	0.35990 (10)	0.0151 (3)	
C6	0.7349 (2)	0.62034 (12)	0.35643 (12)	0.0160 (4)	
H6A	0.6528	0.6212	0.3975	0.019*	
H6B	0.8088	0.6606	0.3679	0.019*	
C7	0.5652 (2)	0.69987 (10)	0.28069 (12)	0.0156 (3)	
H7A	0.6323	0.7432	0.2719	0.019*	
H7B	0.5190	0.7043	0.3340	0.019*	
C8	0.7933 (2)	0.64051 (11)	0.21953 (12)	0.0162 (4)	
H8A	0.8566	0.5952	0.2212	0.019*	
H8B	0.8607	0.6814	0.2372	0.019*	
C9	0.7479 (2)	0.65450 (12)	0.13510 (12)	0.0161 (4)	
C10	0.6559 (2)	0.60220 (10)	0.09514 (11)	0.0155 (4)	
C11	0.6273 (2)	0.61288 (12)	0.01400 (12)	0.0188 (4)	
H11	0.5663	0.5782	−0.0142	0.023*	
C12	0.6873 (3)	0.67359 (13)	−0.02515 (12)	0.0232 (4)	
H12	0.6664	0.6801	−0.0798	0.028*	
C13	0.7777 (3)	0.72492 (13)	0.01476 (14)	0.0251 (4)	
H13	0.8186	0.7663	−0.0123	0.030*	
C14	0.8073 (2)	0.71496 (11)	0.09491 (12)	0.0199 (4)	
H14	0.8688	0.7498	0.1225	0.024*	

### Atomic displacement parameters (Å^2^)

**Table:**

	*U*^11^	*U*^22^	*U*^33^	*U*^12^	*U*^13^	*U*^23^
Tb1	0.01260 (5)	0.01031 (5)	0.01225 (5)	0.000	0.00008 (7)	0.000
O1	0.0177 (6)	0.0180 (6)	0.0145 (6)	−0.0021 (5)	0.0023 (5)	−0.0009 (5)
O2	0.0439 (9)	0.0209 (7)	0.0287 (8)	−0.0086 (7)	−0.0077 (7)	0.0083 (6)
O3	0.096 (2)	0.0096 (8)	0.0704 (19)	0.000	−0.046 (3)	0.000
N1	0.0418 (14)	0.0119 (9)	0.0343 (13)	0.000	−0.028 (2)	0.000
N2	0.0170 (8)	0.0191 (8)	0.0188 (8)	0.0028 (7)	−0.0021 (6)	0.0002 (6)
N3	0.0124 (7)	0.0136 (7)	0.0128 (6)	0.0003 (6)	−0.0002 (5)	0.0006 (5)
C1	0.0244 (10)	0.0208 (10)	0.0294 (11)	0.0046 (8)	−0.0059 (9)	−0.0027 (8)
C2	0.0250 (14)	0.0275 (10)	0.0298 (10)	0.0106 (8)	−0.0037 (8)	0.0042 (8)
C3	0.0180 (13)	0.0364 (11)	0.0214 (9)	0.0023 (8)	−0.0036 (7)	0.0102 (8)
C4	0.0176 (11)	0.0282 (10)	0.0153 (8)	−0.0033 (7)	−0.0028 (6)	0.0061 (8)
C5	0.0147 (8)	0.0196 (9)	0.0110 (8)	0.0001 (7)	0.0013 (6)	0.0032 (7)
C6	0.0162 (9)	0.0181 (9)	0.0138 (9)	−0.0009 (8)	−0.0022 (7)	−0.0010 (8)
C7	0.0161 (8)	0.0110 (8)	0.0198 (8)	−0.0006 (7)	−0.0002 (7)	−0.0012 (7)
C8	0.0124 (8)	0.0210 (9)	0.0153 (8)	−0.0023 (7)	0.0007 (7)	0.0021 (7)
C9	0.0136 (9)	0.0209 (10)	0.0139 (9)	0.0017 (8)	0.0009 (7)	0.0012 (8)
C10	0.0130 (8)	0.0175 (9)	0.0158 (8)	0.0028 (7)	0.0024 (7)	0.0001 (7)
C11	0.0181 (9)	0.0231 (10)	0.0153 (9)	0.0024 (8)	0.0000 (7)	−0.0021 (8)
C12	0.0266 (11)	0.0286 (11)	0.0142 (9)	0.0029 (9)	−0.0004 (8)	0.0042 (8)
C13	0.0303 (11)	0.0238 (10)	0.0210 (11)	−0.0032 (9)	0.0013 (9)	0.0080 (8)
C14	0.0196 (9)	0.0208 (9)	0.0194 (10)	−0.0012 (8)	0.0002 (8)	0.0022 (8)

### Geometric parameters (Å, º)

**Table:**

Tb1—O1^i^	2.1947 (13)	C3—H3	0.9500
Tb1—O1	2.1947 (13)	C4—C5	1.389 (3)
Tb1—O2^i^	2.4764 (15)	C4—H4	0.9500
Tb1—O2	2.4764 (15)	C5—C6	1.513 (3)
Tb1—N2^i^	2.5521 (17)	C6—H6A	0.9900
Tb1—N2	2.5521 (17)	C6—H6B	0.9900
Tb1—N3^i^	2.5558 (16)	C7—C7^i^	1.529 (4)
Tb1—N3	2.5558 (16)	C7—H7A	0.9900
Tb1—N1	2.891 (2)	C7—H7B	0.9900
O1—C10	1.324 (2)	C8—C9	1.503 (3)
O2—N1	1.266 (2)	C8—H8A	0.9900
O3—N1	1.220 (3)	C8—H8B	0.9900
N1—O2^i^	1.266 (2)	C9—C14	1.393 (3)
N2—C1	1.342 (3)	C9—C10	1.412 (3)
N2—C5	1.347 (3)	C10—C11	1.409 (3)
N3—C6	1.485 (2)	C11—C12	1.390 (3)
N3—C7	1.489 (2)	C11—H11	0.9500
N3—C8	1.498 (2)	C12—C13	1.391 (3)
C1—C2	1.391 (3)	C12—H12	0.9500
C1—H1	0.9500	C13—C14	1.392 (3)
C2—C3	1.380 (3)	C13—H13	0.9500
C2—H2	0.9500	C14—H14	0.9500
C3—C4	1.388 (3)		
			
O1^i^—Tb1—O1	154.07 (7)	C8—N3—Tb1	111.56 (11)
O1^i^—Tb1—O2^i^	128.52 (6)	N2—C1—C2	123.4 (2)
O1—Tb1—O2^i^	77.36 (6)	N2—C1—H1	118.3
O1^i^—Tb1—O2	77.36 (6)	C2—C1—H1	118.3
O1—Tb1—O2	128.52 (6)	C3—C2—C1	118.3 (2)
O2^i^—Tb1—O2	51.64 (9)	C3—C2—H2	120.9
O1^i^—Tb1—N2^i^	98.20 (5)	C1—C2—H2	120.9
O1—Tb1—N2^i^	86.89 (5)	C2—C3—C4	119.11 (19)
O2^i^—Tb1—N2^i^	80.45 (6)	C2—C3—H3	120.4
O2—Tb1—N2^i^	79.10 (6)	C4—C3—H3	120.4
O1^i^—Tb1—N2	86.89 (5)	C3—C4—C5	119.2 (2)
O1—Tb1—N2	98.20 (5)	C3—C4—H4	120.4
O2^i^—Tb1—N2	79.10 (6)	C5—C4—H4	120.4
O2—Tb1—N2	80.45 (6)	N2—C5—C4	122.21 (19)
N2^i^—Tb1—N2	157.26 (8)	N2—C5—C6	117.05 (16)
O1^i^—Tb1—N3^i^	76.70 (5)	C4—C5—C6	120.74 (19)
O1—Tb1—N3^i^	82.18 (5)	N3—C6—C5	111.54 (16)
O2^i^—Tb1—N3^i^	141.99 (5)	N3—C6—H6A	109.3
O2—Tb1—N3^i^	132.91 (6)	C5—C6—H6A	109.3
N2^i^—Tb1—N3^i^	66.65 (5)	N3—C6—H6B	109.3
N2—Tb1—N3^i^	135.88 (5)	C5—C6—H6B	109.3
O1^i^—Tb1—N3	82.18 (5)	H6A—C6—H6B	108.0
O1—Tb1—N3	76.70 (5)	N3—C7—C7^i^	113.05 (13)
O2^i^—Tb1—N3	132.91 (6)	N3—C7—H7A	109.0
O2—Tb1—N3	141.99 (5)	C7^i^—C7—H7A	109.0
N2^i^—Tb1—N3	135.88 (5)	N3—C7—H7B	109.0
N2—Tb1—N3	66.65 (5)	C7^i^—C7—H7B	109.0
N3^i^—Tb1—N3	70.67 (7)	H7A—C7—H7B	107.8
O1^i^—Tb1—N1	102.97 (4)	N3—C8—C9	116.63 (16)
O1—Tb1—N1	102.97 (4)	N3—C8—H8A	108.1
O2^i^—Tb1—N1	25.82 (4)	C9—C8—H8A	108.1
O2—Tb1—N1	25.82 (4)	N3—C8—H8B	108.1
N2^i^—Tb1—N1	78.63 (4)	C9—C8—H8B	108.1
N2—Tb1—N1	78.63 (4)	H8A—C8—H8B	107.3
N3^i^—Tb1—N1	144.67 (4)	C14—C9—C10	120.42 (18)
N3—Tb1—N1	144.67 (4)	C14—C9—C8	120.25 (19)
C10—O1—Tb1	139.80 (12)	C10—C9—C8	119.08 (19)
N1—O2—Tb1	95.73 (12)	O1—C10—C11	121.83 (18)
O3—N1—O2	121.55 (11)	O1—C10—C9	120.05 (17)
O3—N1—O2^i^	121.55 (11)	C11—C10—C9	118.13 (18)
O2—N1—O2^i^	116.9 (2)	C12—C11—C10	120.67 (19)
O3—N1—Tb1	180.0	C12—C11—H11	119.7
O2—N1—Tb1	58.45 (11)	C10—C11—H11	119.7
O2^i^—N1—Tb1	58.45 (11)	C11—C12—C13	120.8 (2)
C1—N2—C5	117.83 (17)	C11—C12—H12	119.6
C1—N2—Tb1	126.43 (14)	C13—C12—H12	119.6
C5—N2—Tb1	115.74 (12)	C12—C13—C14	119.2 (2)
C6—N3—C7	109.19 (15)	C12—C13—H13	120.4
C6—N3—C8	107.09 (15)	C14—C13—H13	120.4
C7—N3—C8	111.34 (15)	C13—C14—C9	120.8 (2)
C6—N3—Tb1	105.69 (12)	C13—C14—H14	119.6
C7—N3—Tb1	111.67 (11)	C9—C14—H14	119.6
			
Tb1—O2—N1—O3	180.000 (1)	Tb1—N3—C7—C7^i^	−38.2 (2)
Tb1—O2—N1—O2^i^	0.000 (1)	C6—N3—C8—C9	−179.19 (19)
C5—N2—C1—C2	−0.5 (3)	C7—N3—C8—C9	−59.9 (2)
Tb1—N2—C1—C2	178.82 (17)	Tb1—N3—C8—C9	65.61 (19)
N2—C1—C2—C3	0.2 (4)	N3—C8—C9—C14	125.3 (2)
C1—C2—C3—C4	0.8 (3)	N3—C8—C9—C10	−60.3 (3)
C2—C3—C4—C5	−1.3 (3)	Tb1—O1—C10—C11	−142.18 (16)
C1—N2—C5—C4	0.0 (3)	Tb1—O1—C10—C9	37.5 (3)
Tb1—N2—C5—C4	−179.43 (14)	C14—C9—C10—O1	−179.29 (18)
C1—N2—C5—C6	−179.33 (18)	C8—C9—C10—O1	6.3 (3)
Tb1—N2—C5—C6	1.2 (2)	C14—C9—C10—C11	0.4 (3)
C3—C4—C5—N2	0.9 (3)	C8—C9—C10—C11	−174.00 (18)
C3—C4—C5—C6	−179.80 (18)	O1—C10—C11—C12	179.21 (19)
C7—N3—C6—C5	173.14 (16)	C9—C10—C11—C12	−0.5 (3)
C8—N3—C6—C5	−66.2 (2)	C10—C11—C12—C13	0.3 (3)
Tb1—N3—C6—C5	52.88 (17)	C11—C12—C13—C14	−0.2 (4)
N2—C5—C6—N3	−38.3 (2)	C12—C13—C14—C9	0.1 (3)
C4—C5—C6—N3	142.41 (18)	C10—C9—C14—C13	−0.2 (3)
C6—N3—C7—C7^i^	−154.7 (2)	C8—C9—C14—C13	174.1 (2)
C8—N3—C7—C7^i^	87.2 (2)		

Symmetry code: (i) −*x*+1, *y*, −*z*+1/2.

### Hydrogen-bond geometry (Å, º)

**Table:**

*D*—H···*A*	*D*—H	H···*A*	*D*···*A*	*D*—H···*A*
C8—H8*B*···O3^ii^	0.99	2.37	3.338 (3)	166

Symmetry code: (ii) *x*+1/2, *y*+1/2, *z*.

## References

[bb1] Anjos, A. dos, Bortoluzzi, A. J., Caro, M. S. B., Peralta, R. A., Friedermann, G. R., Mangrich, A. S. & Neves, A. (2006). *J. Braz. Chem. Soc.* **17**, 1540–1550.

[bb2] Bruker (2010). *APEX2* and *SAINT*. Bruker AXS Inc., Madison, Wisconsin, USA.

[bb3] Bruker (2014). *SADABS*. Bruker AXS Inc., Madison, Wisconsin, USA.

[bb4] Chilton, N. F., Langley, S. K., Moubaraki, B., Soncini, A., Batten, S. R. & Murray, K. S. (2013). *Chem. Sci.* **4**, 1719–1730.

[bb5] Colotti, G., Ilari, A., Boffi, A. & Morea, V. (2013). *Mini Rev. Med. Chem.* **13**, 211–221. 23438056

[bb6] Diehl, H., Hach, C. C. & Bailar, J. C. (2007). *Inorganic Synthesis*, pp. 196–201. New York: John Wiley & Sons Inc.

[bb7] Farrugia, L. J. (2012). *J. Appl. Cryst.* **45**, 849–854.

[bb8] Golchoubian, H. & Gholamnezhad, P. (2009). *X-Ray Struct. Anal. Online*, **25**, 95–96.

[bb9] Hancock, R. D. (2013). *Chem. Soc. Rev.* **42**, 1500–1524.

[bb10] Happe, T. & Hemschemeier, A. (2014). *Trends Biotechnol.* **32**, 170–176. 10.1016/j.tibtech.2014.02.004 24630475

[bb11] Johnson, C. K. (1976). *ORTEPII*. Report ORNL-5138. Oak Ridge National Laboratory, Tennessee, USA.

[bb12] Lanznaster, M., Neves, A., Bortoluzzi, A. J., Assumpção, A. M. C., Vencato, I., Machado, S. P. & Drechsel, S. M. (2006). *Inorg. Chem.* **45**, 1005–1011. 10.1021/ic050869o 16441107

[bb13] Lenze, M., Sedinkin, S. L. & Bauer, E. B. (2013). *J. Mol. Catal. A Chem.* **373**, 161–171.

[bb14] Luzon, J. & Sessoli, R. (2012). *Dalton Trans.* **41**, 13556–13567. 10.1039/c2dt31388j 22936346

[bb15] Neves, A., Ceccato, A. S., Erthal, S. M. D., Vencato, I., Nuber, B. & Weiss, J. (1991). *Inorg. Chim. Acta*, **187**, 119–121.

[bb16] Neves, A., Ceccatto, A. S., Erasmus-Buhr, C., Gehring, S., Haase, W., Paulus, H., Nascimento, O. R. & Batista, A. A. (1993). *J. Chem. Soc. Chem. Commun.* pp. 1782–1784.

[bb17] Neves, A., Erthal, S. M. D., Vencato, I., Ceccato, A. S., Mascarenhas, Y. P., Nascimento, O. R., Horner, M. & Batista, A. A. (1992). *Inorg. Chem.* **31**, 4749–4755.

[bb18] Nurchi, V. M., Crespo-Alonso, M., Toso, L., Lachowicz, J. I. & Crisponi, G. (2013). *Mini Rev. Med. Chem.* **13**, 1541–1549. 10.2174/13895575113139990077 23895193

[bb19] Parsons, S. & Flack, H. (2004). *Acta Cryst.* A**60**, s61.

[bb20] Rinehart, J. D. & Long, J. R. (2011). *Chem. Sci.* **2**, 2078–2085.

[bb21] Schwingel, E. W., Arend, K., Zarling, J., Neves, A. & Szpoganicz, B. (1996). *J. Braz. Chem. Soc.* **7**, 31–37.

[bb22] Sears, M. E. (2013). *Sci. World J.*, article no. 219840.

[bb23] Sessoli, R. & Powell, A. K. (2009). *Coord. Chem. Rev.* **253**, 2328–2341.

[bb24] Setyawati, I. A., Liu, S., Rettig, S. J. & Orvig, C. (2000). *Inorg. Chem.* **39**, 496–507. 10.1021/ic991170j 11229569

[bb25] Sheldrick, G. M. (2008). *Acta Cryst.* A**64**, 112–122. 10.1107/S0108767307043930 18156677

[bb26] Thomas, F., Arora, H., Philouze, C. & Jarjayes, O. (2010). *Inorg. Chim. Acta*, **363**, 3122–3130.

[bb27] Totaro, P., Westrup, K. C. M., Boulon, M.-E., Nunes, G. G., Back, D. F., Barison, A., Ciattini, S., Mannini, M., Sorace, L., Soares, J. F., Cornia, A. & Sessoli, R. (2013). *Dalton Trans.* **42**, 4416–4426. 10.1039/c2dt32618c 23280320

[bb28] Ungur, L., Le Roy, J. J., Korobkov, I., Murugesu, M. & Chibotaru, L. F. (2014). *Angew. Chem. Int. Ed.* **53**, 4413–4417. 10.1002/anie.201310451 24652777

[bb29] Westrup, K. C. M., Boulon, M.-E., Totaro, P., Nunes, G. G., Back, D. F., Barison, A., Jackson, M., Paulsen, C., Gatteschi, D., Sorace, L., Cornia, A., Soares, J. F. & Sessoli, R. (2014). *Chem. Eur. J.* **20**, 13681–13691. 10.1002/chem.201403361 25200792

[bb30] Wong, E., Caravan, P., Liu, S., Rettig, S. J. & Orvig, C. (1996). *Inorg. Chem.* **35**, 715–724.

[bb31] Wong, E., Liu, S., Rettig, S. & Orvig, C. (1995). *Inorg. Chem.* **34**, 3057–3064.

[bb32] Xu, L., Setyawati, I. A., Pierreroy, J., Pink, M., Young, V. G., Patrick, B. O., Rettig, S. J. & Orvig, C. (2000). *Inorg. Chem.* **39**, 5958–5963. 10.1021/ic001273y 11188520

[bb33] Yamada, Y., Takenouchi, S. I., Miyoshi, Y. & Okamoto, K. I. (2010). *J. Coord. Chem.* **63**, 996–1012.

[bb34] Zhang, P., Zhang, L., Wang, C., Xue, S. F., Lin, S. Y. & Tang, J. K. (2014). *J. Am. Chem. Soc.* **136**, 4484–4487. 10.1021/ja500793x 24625001

